# Object location learning in mice requires hippocampal somatostatin interneuron activity and is facilitated by mTORC1-mediated long-term potentiation of their excitatory synapses

**DOI:** 10.1186/s13041-022-00988-7

**Published:** 2022-12-21

**Authors:** Eve Honoré, Jean-Claude Lacaille

**Affiliations:** Department of Neurosciences, Center for Interdisciplinary Research on Brain and Learning (CIRCA) and Research Group on Neural Signaling and Circuitry (GRSNC), Université de Montréal, P.O. Box 6128, Station Downtown, QC H3C 3J7 Montreal, Canada

**Keywords:** Dorsal CA1 hippocampus, Somatostatin interneurons, Long-term potentiation, Object location memory, mTORC1, Optogenetic silencing, Optogenetic synaptic plasticity

## Abstract

Hippocampus-dependent learning and memory originate from long-term synaptic changes in hippocampal networks. The activity of CA1 somatostatin interneurons (SOM-INs) during aversive stimulation is necessary for contextual fear memory formation. In addition, mTORC1-dependent long-term potentiation (LTP) of SOM-IN excitatory input synapses from local pyramidal cells (PC-SOM synapses) contributes to the consolidation of fear motivated spatial and contextual memories. Although, it remains unknown if SOM-IN activity and LTP are necessary and sufficient for novelty motivated spatial episodic memory such as the object location memory, and if so when it is required. Here we use optogenetics to examine whether dorsal CA1 SOM-IN activity and LTP are sufficient to regulate object location memory. First, we found that silencing SOM-INs during object location learning impaired memory. Second, optogenetic induction of PC-SOM synapse LTP (TBS_opto_) given 30 min before object location training, resulted in facilitation of memory. However, in mice with mTORC1 pathway genetically inactivated in SOM-INs, which blocks PC-SOM synapse LTP, TBS_opto_ failed to facilitate object location memory. Our results indicate that SOM-IN activity is necessary during object location learning and that optogenetic induction of PC-SOM synapse LTP is sufficient to facilitate consolidation of object location memory. Thus, hippocampal somatostatin interneuron activity is required for object location learning, a hippocampus-dependent form of novelty motivated spatial learning that is facilitated by plasticity at PC-SOM synapses.

**Supplementary Information:**

The online version contains supplementary material available at 10.1186/s13041-022-00988-7.

## Introduction

Hippocampal somatostatin expressing interneurons (SOM-INs) are dendrite-projecting inhibitory cells [1]. The activity of CA1 SOM-INs is necessary for the proper formation of contextual fear memory [2, 3]. SOM-INs may also be critical during place cell firing by controlling pyramidal cell excitatory inputs, limiting dendritic amplification and suppressing out-of-field excitatory inputs [4, 5]. The contribution of SOM-INs has been studied particularly in the dorsal part of CA1, in relation to the encoding of spatial and contextual memory by PCs. However, the contribution to aversive episodic memory of a subset of SOM-INs, the oriens lacunosum-moleculare interneurons that express the nicotinic a2 subunit (OLMa2), varies depending on their location along the dorsoventral axis of the hippocampus, with activation of cells in intermediate hippocampus inhibiting fear-related memories [6].

A large body of evidence suggests that long-term synaptic plasticity between hippocampal principal cells is a fundamental substrate supporting memory [7–11]. Inhibitory interneuron synapses also display a wide range of plasticity [1, 12, 13]. Notably, SOM-INs display an input-specific long-term potentiation (LTP) at their main excitatory inputs coming from local pyramidal cells (PC; PC-SOM synapses) [14–16]. This LTP is Hebbian and depends on the activation of the metabotropic glutamate receptor subtype 1a (mGluR1a) and mechanistic target of rapamycin complex 1 (mTORC1) pathways [14, 15, 17–19]. PC-SOM synapse LTP is triggered by theta burst stimulation (TBS) of PCs or contextual fear conditioning and is absent in parvalbumin expressing interneurons or in interneurons of stratum radiatum [3, 17–19]. PC-SOM synapse LTP in turn regulates CA1 network metaplasticity at CA3 and entorhinal inputs to PCs [3, 15, 18, 20]. Besides, PC-SOM synapse LTP is required during learning for the consolidation of contextual fear memory [3, 19]. Moreover, SOM-IN activity after contextual fear conditioning supports memory consolidation [20]. SOM-IN mTORC1 dependent plasticity also contributes to fear motivated spatial memory, as in the Barnes maze, in which mice need to learn over repeated trials the location of a target hole to escape a bright, loud, open environment [19]. Thus, SOM-IN activity and PC-SOM synapse LTP during learning and consolidation, control the formation of fear-related contextual and spatial memories.

Hippocampal synaptic plasticity also plays an important role in novelty driven learning, particularly in the processing of novel object spatial configuration [21–23]. Novel spatial learning such as spatial object recognition triggers long-term depression (LTD) that curtails LTP at hippocampal PC synapses [21, 24]. Hippocampal PC LTD creates a neuronal representation of spatial content by eliminating weakly potentiated synapses and by dictating temporal constraints and the dendritic distribution of LTP [25]. Therefore, the hippocampus recognizes and encodes the novel spatial component of an object through LTD and metaplastic changes at PC synapses [21, 23, 25].

The activity of SOM-IN from dorsal CA1 is not required for the encoding of object shape while inhibiting intermediate CA1 SOM-INs during learning facilitates later novel object recognition [6]. The object location memory task, which largely depends on dorsal CA1 function, is a one trial, novelty driven, spatial episodic memory test [26–29]. Given the role of PC-SOM synapse LTP in spatial memory [19] and the dependency of object location memory task on dorsal CA1 [26–29], we asked if dorsal CA1 SOM-IN activity and PC-SOM synapse LTP contribute to the encoding of such non-aversive forms of spatial episodic memory.

To address this question, we used optogenetics to silence SOM-IN activity during training of an object location memory task. We found that silencing dorsal hippocampal SOM-INs during training impaired object location memory tested 24 h later. Furthermore, optogenetic induction of PC-SOM synapse LTP given 30 min before training resulted in facilitation of object location memory tested 24 h later. This facilitation was absent in mice with conditional knock-out of *Rptor* in SOM-INs which lack PC-SOM synapse LTP. Hence, our results indicate that SOM-IN activity is necessary during object location learning and that optogenetic induction of PC-SOM synapse LTP is sufficient to facilitate consolidation of object location memory. These findings shed new light on the essential role of activity of SOM-INs in novelty-driven spatial episodic memory that is facilitated by plasticity at PC-SOM synapses.

## Methods 

### Animals

Animal procedures and experiments were performed in accordance with the Université de Montréal Animal Care Committee (Comité de Déontologie de l’Expérimentation sur les Animaux; CDEA Protocols # 17-001, 17-002, 18-002, 18-003, 19-003, 19-004, 20-001, 20-002, 21-001, 21-002, 22-008, 22-009) and the Canadian Council of Animal Care guidelines.

Mice with Cre-dependent Enhanced Yellow Fluorescent Protein (EYFP) expression in SOM-INs (SOM-Cre-EYFP mice) were produced by crossing Sst^ires−Cre^ mice (Jackson Labs #013044) with *Rosa26*^lsl−EYFP^ reporter mice (Ai3, Jackson Labs # 007903) as previously [3, 19]. Mice with a conditional homozygous knock-out of *Rptor* in SOM-INs (SOM-Cre-Raptor-KO mice) were obtained by crossing Sst^ires−Cre^; *Rosa26*^lsl−EYFP^ with Rptor^fl/fl^ mice as previously [3, 19]. Mice were housed 2–4 animals per cage, until optogenetic cannula implantation after which they were singly housed in larger cages (rat cage) with some object enrichment (small plastic weight boat and PVC tube). Food and water were given ad libitum. Mice were maintained on a 12 h light/dark cycle. Experiments were performed during the light phase on male mice.

### Virus injection, optic fiber implantation and optogenetic stimulation

Six to eight weeks old mice were anesthetized with intraperitoneal injection of ketamine (50 mg/kg) and xylazine (5 mg/kg). For experiments with SOM-IN silencing, 0.5 µL of AAV2/9-flex-Arch-GFP (1.74–1.83 × 10^13^ particles/ml) or AAV2/9-EF1a-DIO-EYFP (3.95 × 10^13^ particles/ml) were injected bilaterally (0.05 µL/min) in dorsal CA1 hippocampus (coordinates relative to bregma: − 1.95 mm AP; ± 1.30 mm ML; and − 1.30 mm DV) as previously [3]. For experiments with TBS optogenetic induction, 0.5 µL of AAV2/9-CaMKIIa-hChR2(E123T/T159C)-mCherry (1.5–1.77 × 10^12^ particles/ml) was injected as described above. After a recovery period of one week, mice were re-anesthetized as described above, and optic fiber cannulas (multimode optic fiber FT200EMT: 0.39 NA, Low OH, 200 µm core diameter; Thorlabs) were implanted bilaterally to target dorsal CA1 hippocampus just above stratum oriens (coordinates relative to bregma: -1.95 mm AP; ± 1.60 mm ML; and − 1.15 mm DV) and sealed with dental cement mixed with carbon powder (Metabond, Parkell Inc). Mice were allowed to rest for a week before behavioral experiments.

For in vivo optogenetic stimulation, a Quadruple Laser Diode Fiber Light Source (LDFLS_405_450_520_638; Doric Lenses Inc) was coupled to Mono Fiberoptic Patchcords (MFP_200/240/900-0.22_2m_FC-ZF1.25(F), 200 µm Core diameter, 0.22 NA; Doric Lenses Inc) and hand-made fiber optic cannulas (optic fiber: FT200EMT, 200 µm Core diameter, 0.39 NA; ceramic ferrule: CFLC230-10; Thorlabs).

### Behavioral experiments 

For the SOM-IN silencing experiments, mice had 1 week recovery between viral injection and cannula implantation. Handling started after 2 weeks recovery (9–11 weeks old mice). For the optogenetic LTP experiments, mice underwent viral injection and cannula implantation the same day. Handling started after 1 week recovery (7–9 weeks old mice). Before each experimental session, the arena and objects were thoroughly cleaned with a 10% Versaclean solution.

#### Handling

Animals were handled for 5 days (5 min/day) and progressively habituated to contention and optic fibers connection (without light stimulation).

#### Open field test

On the last day of habituation, mice were allowed to freely explore a square arena (45 × 45 cm; Panlab) for 10 min. The anxiety level was assessed by measuring the time spent and the distance traveled in the center (1/3 central zone) and the periphery (1/3 peripheral zone) of the arena. Locomotion was assessed by measuring the total distance traveled and the number of zone transitions (16 equal square zones). Mice were video-tracked and their movements analyzed using a position tracking system (Smart 3.0; PanLab). Animals showing abnormal levels of anxiety in the open field test were excluded from the study.

#### Object location memory task

The task consisted of four days of habituation, one day of object location training and one day of object location memory test. For this task, the square arena (45 × 45 cm; Panlab) had three uniform light gray walls and one differentiated wall with a glossy black square in the center (1/3 of the wall). For habituation, mice were connected to the patch cord either immediately before entering the arena for experiments with SOM-IN silencing, or 30 min before in the home cage for the experiments with TBS optogenetic induction. Mice were allowed to explore the empty arena for 5 min a day.

For object location training**,** two identical objects (aluminum post with black plastic star grip screw on top; Siskiyou Design-Instruments) were placed 10 cm from the differentiated wall, each in line with one end of the glossy black square. For SOM-IN silencing experiments, the animals were attached to the patch cord, placed in the arena and allowed to explore the arena and objects for 10 min. SOM-INs were silenced during the complete training session with continuous 520 nm light stimulation. For experiments with optogenetic TBS (TBS_opto_) induction in the home cage before training, mice were given the TBS_opto_ protocol (450 nm; 5 bursts of 4 light pulses of 1 ms duration at 80 Hz, given at 300 ms interburst interval, and repeated 3 times with 30 s interval), or no light stimulation. Thirty minutes later, mice were allowed to explore the arena and objects for 10 min. For object location memory test 24 h later, one object was moved to a new location across the arena, 10 cm from the opposite wall. Mice were placed in the arena and allowed to explore the arena and objects for 10 min.

Object location experiments were carried out using 2 contiguous arenas. The allocation to arena 1 or 2, and the object to be moved during the test (right or left), were counterbalanced between mice and groups. All sessions were video tracked at 30 frame/s (1080 pixels). Behavior was analyzed using DeepLabCut [30], as follows. The deep neural network was trained to detect the head, body, and tail base of the mice, as well as the object that stayed at the original place (immobile object) and the object that moved for the test session (mobile object), on a randomly generated sample of frames taken from memory acquisition and test videos from each batch of experiments (≥ 20 frames per videos, > 200 frames per batches). The object exploration time was measured as the time the mouse’s head was in a 3 cm zone around each object (see analysis code in Additional file 2). ResNet50 deep neural network was trained until tests and trained errors where approximately 3 pixels. All analyzed videos were watched by the experimenter to ensure there was no failure to detect object exploration. The exploration time for each object was expressed as a percentage of total object exploration time: (time exploring mobile object)/(time exploring mobile object + immobile object) × 100. The object preference ratio was calculated as: (time exploring mobile object)/(time exploring immobile object). Mice were excluded if the discrimination index (time exploring mobile object − time exploring immobile object)/(time exploring mobile object + time exploring immobile object) during training was not between − 30 and 30, if the time of exploration of one object was ≤ 3 s, or if total exploration time was ≤ 10 s during the test [31].

### Histology

After behavioral experiments, animals were deeply anesthetized with intraperitoneal injection of sodium pentobarbital (MTC Pharmaceuticals), trans-cardially perfused first with 0.1 M phosphate buffer saline (PBS) and next with 4% paraformaldehyde diluted in 0.1 M PBS (PFA). The brains were isolated, postfixed in 4% PFA for 24 h and cryoprotected in 30% sucrose. Coronal brain sections (50 µm thick) were obtained using freezing microtome (SM200R; Leica) and mounted in ProLong Gold (Invitrogen). Immunofluorescence was used to enhance GFP fluorescence associated with Arch. Brain sections of mice injected with AAV9.flex.CBA.Arch-GFP.WPRE.SW40 were prepared as described above, permeabilized with 0.5% Triton X-100 in PBS for 30 min and unspecific binding was blocked with 10% normal goat serum in 0.1% Triton X-100/PBS for 1 h. Rabbit polyclonal anti GFP (1/200) antibodies were incubated 24 h at 4 °C. Sections were subsequently incubated at room temperature with Alexa Fluor 594-conjugated goat anti-rabbit IgGs (1/500; 60 min). Sections were mounted in ProLong Gold (Invitrogen), examined using an epifluorescence microscope (Eclipse E600; Nikon) and images were acquired with the Simple PCI software. Mice were excluded if virus expression was not restricted to dorsal CA1 hippocampus, or optic fiber placement was not correct (just above stratum oriens of dorsal CA1 hippocampus).

### Statistical analysis

Statistical analysis was performed using SigmaPlot (Systat Software) or SPSS (IBM Statistics). Data were tested for normality and equal variability. Mann–Whitney Rank Sum tests and Kruskal–Wallis one-way ANOVA on Ranks with Dunn’s pairwise comparisons were used when data did not pass normality and/or homoscedasticity assumptions. Otherwise, Student’s t-tests, paired t-test, one-way ANOVA, or 2-repeated measures ANOVA followed by Bonferroni pairwise comparisons, were used. All tests were two-sided. All data in the Figures are presented as mean ± SEM. Box plots in Figures represent mean, 25^th^ and 75^th^ percentiles, and ± SEM. Asterisks in Figures denote statistical significance levels for specified tests (*p < 0.05; **p < 0.01; ***p < 0.005; ns, not significant). Sample size required to reach significance were determined with a power analysis with power = 0.8 and alpha = 0.05. Details of every statistical test are listed in Additional file 1 and identified per Figure panel.

## Results

### Somatostatin interneuron activity is necessary during object location learning

**Fig. 1:**
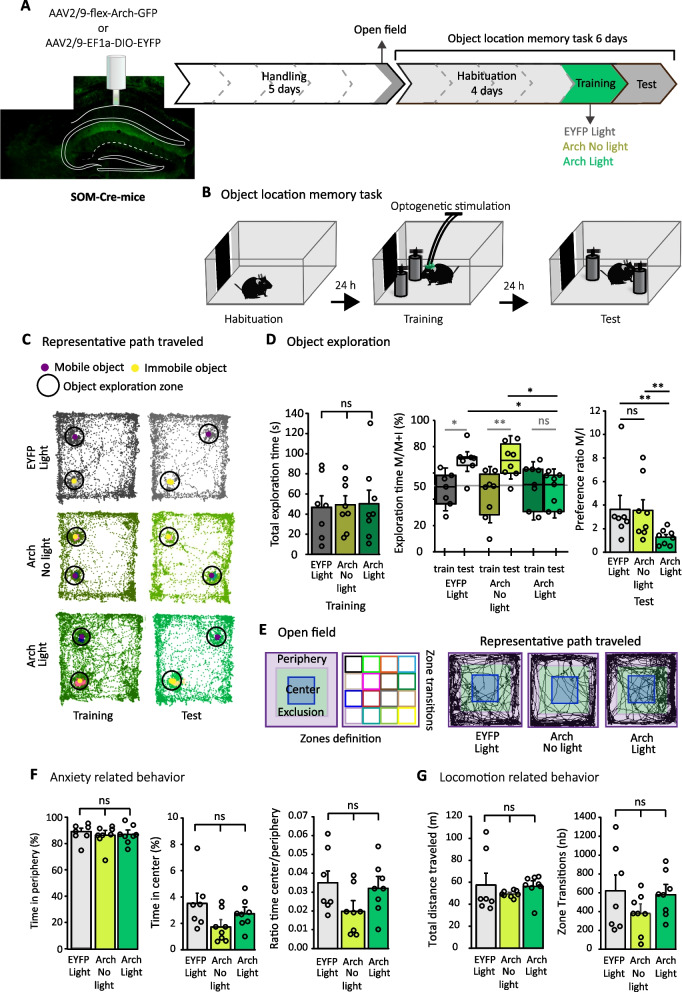
Somatostatin interneuron activity is necessary during object location memory acquisition. **A** Left: representative image of CAG-driven cre-dependent Arch-GFP expression in a SOM-Cre mice. Right: diagram of behavioral testing sequence. **B** Experimental protocol of the object location memory task with optogenetic light stimulation (520 nm) for the duration of the training session. **C–G** Color coding of groups is as follows. Grey: EYFP injected mice with light, n = 7 mice. Chartreuse green: Arch injected mice without light (Arch No light), n = 8 mice. Emerald green: Arch injected mice with light (Arch Light) n = 8 mice. Dark colors, training; light colors, test. **C** Representative path traveled during training and test sessions. **D** Left: graph of the total exploration time of objects during the training session, showing no difference between groups (one way ANOVA). Middle: graph of the percentage of time spent exploring the mobile object during training and test sessions, showing no difference during training sessions, but reduced exploration time during test session for mice with SOM-INs silencing (Arch Light) relative to controls. Two-way repeated measures ANOVA, Bonferroni pairwise multiple comparisons. Right: graph of preference ratio indicating a deficit in mice with SOM-INs silencing (Arch Light) relative to control mice (EYFP and Arch No light) (Kruskal–Wallis one-way ANOVA on ranks). ns p > 0.05, *p < 0.05, **p < 0.01. **E** Open field. Left: zone separations used for analysis. Right: representative path traveled during the open field test for EYFP, Arch No light and Arch Light mice. **F** Graphs showing similar times spent in periphery (left) or center (middle), and ratio of time in center/periphery (right), indicating normal anxiety. One-way ANOVA, ns p > 0.05. **G** Graphs showing similar total distance traveled (left) and zone transitions (right) in all groups. Total distance: Kruskal–Wallis one-way ANOVA on Ranks. Zone transitions: one-way ANOVA, ns p > 0.05. Details of all statistical tests in this and following figures are listed in Additional file 1

Activity of dorsal CA1 SOM-INs is necessary during the presentation of aversive stimuli for the formation of contextual fear memory [2, 3]. To determine if the activity of SOM-INs is also necessary during learning for object location memory, we used optogenetic inhibition of CA1 SOM-INs. The light-gated proton pump archaerhodopsin (Arch) was expressed in SOM-INs using bilateral injections of AAV2/9-flex-Arch-GFP to the dorsal CA1 region of SOM-Cre mice and control mice received injections of AAV2/9-EF1a-DIO-EYFP (Fig. 1A) [3, 18]. In all experiments, post-hoc histology showed selective expression of Arch-GFP in CA1 SOM interneurons (ex. Figure 1A), as previously [3].

The object location memory task consisted of a training during which the animal was exposed to two identical new objects in an environment, and a memory test 24 h later when the animal was exposed to the same environment but with one object moved to a new location (Fig. 1B, C). The time spent exploring each object was measured and object location memory assessed as the preference for exploration of the object with a novel location in the memory test [31]. During training, silencing SOM-INs by optogenetic activation of Arch (Arch Light) did not affect total exploration time relative to control mice with light stimulation of EYFP expressing SOM-INs (EYFP Light) or without light activation of Arch expressing SOM-INs (Arch No light) (Fig. 1D left). Also, control mice did not show preference for exploration of any object and silencing SOM-INs did not affect the percent exploration time of the mobile object (Fig. 1D middle). Thus, silencing SOM-INs did not affect object exploration during training.

However silencing SOM-INs during training affected object location memory tested 24 h later. Control mice with light stimulation of EYFP expressing SOM-INs during training, or without light stimulation of Arch expressing SOM-INs, spent more time exploring the mobile object during the memory test, but mice with SOM-IN silencing during training did not (Fig. 1D middle). The preference ratio of mobile/immobile object was elevated in both control mice but not in mice with silencing of SOM-INs (Fig. 1D right). Thus, silencing SOM-INs during training results in impairment of object location memory, indicating that activity of SOM-INs supports object location learning.

The effects of silencing SOM-INs were not due to changes in anxiety levels or locomotion since in open field tests (Fig. 1E–G), mice from the 3 groups showed equivalent anxiety levels (% time spent in periphery or center, and ratio of time in center/periphery; Fig. 1F) or locomotion (total distance traveled and zone transitions; Fig. 1G). Thus, dorsal CA1 SOM-IN activity is necessary during learning for formation of object location memory.

### Object location memory is facilitated by optogenetic induction of long-term potentiation of SOM interneuron excitatory afferents

**Fig. 2:**
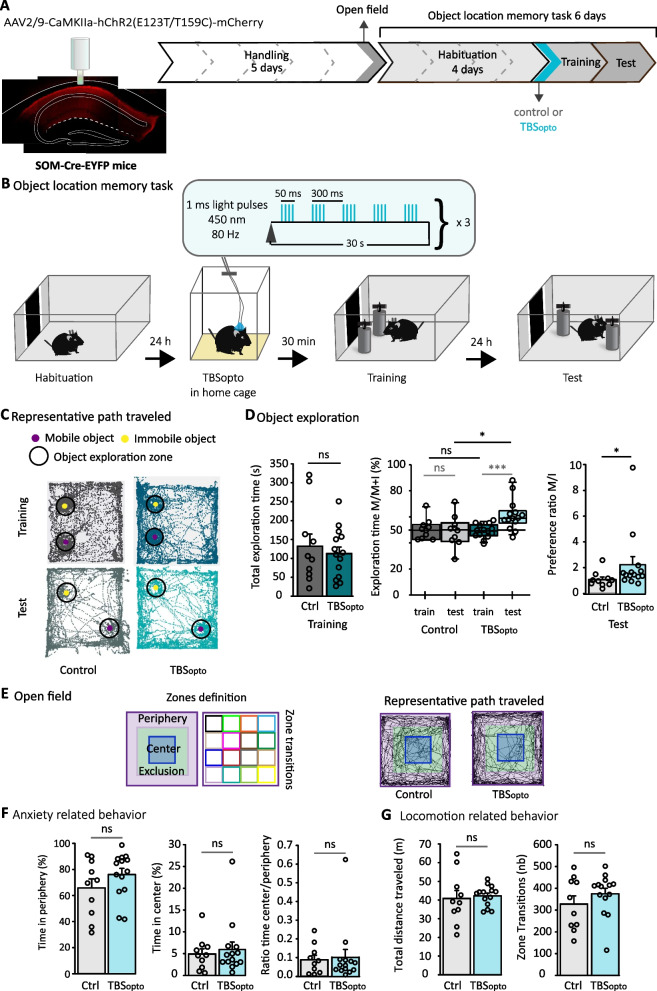
Object location memory is facilitated by optogenetic induction of long-term potentiation of SOM-IN excitatory afferents. **A** Left: schematic of cannulation with representative image of hChR2-mCherry expression of the AAV2/9-CaMKIIa-hChR2(E123T/T159C)-mCherry in dorsal CA1 hippocampus of SOM-Cre-EYFP mice. Right: diagram of behavioral testing sequence (open field and object location memory task). **B** Experimental protocol of the object location memory task with optogenetic induction of PC-SOM synapse LTP (TBS_opto_) 30 min before the training session. **C–G** Color coding of groups is as follows. Grey: control mice without TBS_opto_, n = 10 mice. Blue: mice with TBS_opto_, n = 14 mice. Dark colors = training, light colors = test. **C** Representative path traveled during training and test sessions. **D** Left: graph of total exploration time of objects during the training session, showing no difference between groups (t-test). Middle: graph of the percentage of time spent exploring the mobile object during training and test sessions, showing no difference during training sessions, but increased exploration time during test session for mice that received TBS_opto_ before training, relative to controls. Two-way repeated measures ANOVA, Bonferroni pairwise multiple comparisons. Right: graph of preference ratio showing facilitation of object location memory for mice that received TBS_opto_ relative to control mice without TBS_opto_ (Mann-Witney Rank Sum Test). ns p > 0.05, *p < 0.05, ***p < 0.005. **E** Open field. Left: zone separations used for analysis. Right: representative path traveled during the open field test for control and mice receiving TBS_opto_. **F** Graphs showing similar time spent in periphery (left) or center (middle), and ratio of time in center/periphery (right), indicating normal anxiety in both groups. Mann-Witney Rank Sum Tests, ns p > 0.05. **G** Graphs showing similar total distance traveled (left) and zone transitions (right) in both groups, indicating normal locomotion. Total distance: Mann-Witney Rank Sum Test. Zone transitions: t-test. ns p > 0.05

mTORC1-dependent LTP is induced at PC-SOM synapses by contextual fear learning and contributes to fear motivated contextual and spatial memories [19]. Optogenetic theta burst stimulation (TBS_opto_) of CA1 PCs induces mGluR1a- and mTORC1-mediated LTP at PC-SOM synapses, and TBS_opto_ given in vivo 30 min before contextual fear conditioning negatively regulates contextual fear memory [3]. Thus, we next determined if optogenetic induction of PC-SOM synapse LTP also regulates spatial episodic memory in object location memory task. Modified hChR2 was expressed in CA1 PCs using bilateral injections of AAV2/9-CaMKIIa-hChR2 (E123T/T159C)-mCherry in dorsal CA1 region of SOM-Cre-EYFP mice, and TBS_opto_ protocol (450 nm; 5 bursts of 4 light pulses of 1 ms duration at 80 Hz, given at 300 ms interburst interval, and repeated 3 times with 30 s interval) was delivered in CA1 region, as previously [3] (Fig. 2A, B). In all experiments, post-hoc histology showed selective expression of hChR2-mCherry in CA1 pyramidal cells (ex. Fig. 2A), as previously [3].

TBS_opto_ given 30 min prior to training did not affect total exploration time of objects during training, relative to control mice that did not receive TBS_opto_ (Fig. 2C, D). During training, control mice did not show preference for exploration of any object and TBS_opto_ did not affect the percent exploration time of the mobile object (Fig. 2D middle). Thus, TBS_opto_ given 30 min prior to learning did not affect object exploration during training.

However, TBS_opto_ given prior to training affected object location memory tested 24 h later. In these experiments, modifications of the surgical timeline and behavioral paradigm (see methods) resulted in subthreshold learning of the object location memory task in control mice in our conditions. Although the discrepancy with the control mice showing significant learning in the SOM-IN inhibition experiments (Fig. 1D) prevented us to make comparisons between these experimental cohorts, it allowed us to test whether TBS_opto_ could facilitate object location learning under conditions that were subthreshold for learning. During memory tests, control mice did not spend more time exploring the mobile object, but mice given TBS_opto_ showed increased exploration of the mobile object (Fig. 2D middle). Preference ratio of mobile/immobile object was elevated in mice that received TBS_opto_ relative to control mice (Fig. 2D right). Thus, TBS_opto_ given 30 min prior to training results in facilitation of object location memory, suggesting that induction of synaptic plasticity at PC-SOM synapses facilitates object location learning.

Effects of TBS_opto_ were not due to changes in anxiety levels or locomotion since in open field tests (Fig. 2E–G), mice from both groups showed equivalent anxiety levels (Fig. 2F) or locomotion (Fig. 2G). Thus, optogenetic induction of synaptic plasticity at PC-SOM synapses prior to learning may facilitate the formation of object location memory.

### Conditional knock-out of *Rptor* in SOM interneurons prevents facilitation of object location memory by TBS_opto_

LTP induced by TBS_opto_ at PC-SOM synapses and its regulation of CA1 network metaplasticity and contextual fear memory are blocked by conditional knock-out of *Rptor* in SOM-INs [3]. Although conditional knockout may have occurred in a minority of CA3 pyramidal cells in the SOM-Cre-Raptor-KO mice [32], we have previously shown that the Cre expression in these mice is highly selective to SOM interneurons [18] and that conditional knockout is specific to SOM interneurons and does not affect significantly hippocampal principal cells [19]. Therefore, we tested next if the facilitation of object location memory by TBS_opto_ was due to mTORC1 signaling in SOM-INs, by using the same TBS_opto_ protocol and expressing hChR2 in CA1 pyramidal cells of mice with a conditional knock-out of *Rptor* in SOM-INs (SOM-Cre-Raptor-KO mice) in which TBS_opto_-induced plasticity in SOM-INs is blocked [3].

**Fig. 3:**
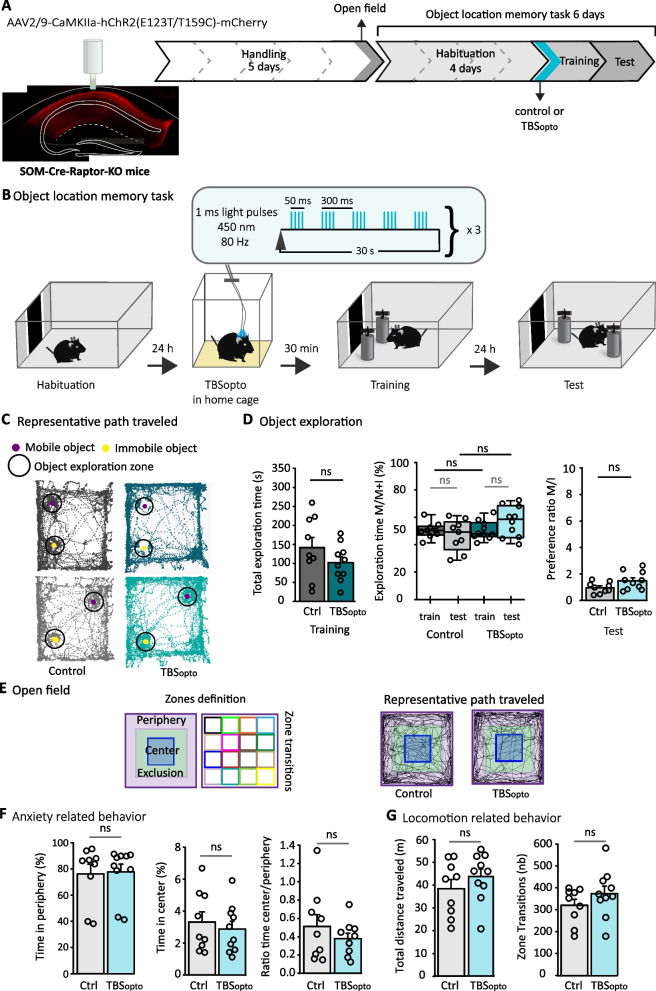
Conditional knock-out of *Rptor* in SOM-INs prevents facilitation of object location memory by TBS_opto_. **A** Left: schematic of cannulation with representative image of hChR2-mCherry expression of the AAV2/9-CaMKIIa-hChR2(E123T/T159C)-mCherry in dorsal CA1 hippocampus of SOM-Cre-Raptor-KO mice. Right: diagram of behavioral testing sequence (open field and object location memory task). **B** Experimental protocol of the object location memory task with TBS_opto_ 30 min before the training session. **C–G** Color coding of groups is as follows. Grey: control mice without TBS_opto_, n = 9 mice. Blue: mice with TBS_opto_, n = 10 mice. Dark colors = training, light colors = test. **C** Representative path traveled during training and test sessions. **D** Left: graph of total exploration time of objects during the training session, showing no difference between groups (t-test). Middle: graph of the percentage of time spent exploring the mobile object during training and test sessions, showing no difference during training and test sessions in both groups, indicating no facilitation of object location memory by TBS_opto_ in SOM-Cre-Raptor-KO mice. Two-way repeated measures ANOVA, Bonferroni pairwise multiple comparisons. Right: graph of preference ratio showing absence of facilitation of object location memory for SOM-Cre-Raptor-KO mice that received TBS_opto_ (t-test). ns p > 0.05. **E** Open field. Left: zone separations used for analysis. Right: representative path traveled during the open field test for control and mice receiving TBS_opto_. **F** Graphs showing similar time spent in periphery (left) or center (middle), and ratio of time in center/periphery (right), indicating normal anxiety in both groups. Time in periphery and ratio, Mann-Witney Rank Sum Tests. Time in center, t-test. ns p > 0.05. **G** Graphs showing similar total distance traveled (left) and zone transitions (right) in both groups, indicating normal locomotion (t-tests). ns p > 0.05

TBS_opto_ given 30 min prior to training in SOM-Cre-Raptor-KO mice did not affect total exploration time of objects during training, relative to control mice without TBS_opto_ (Fig. 3C, D). During training, control mice did not show preference for exploration of any object and TBS_opto_ did not affect the percent exploration time of the mobile object (Fig. 3D middle). Thus, TBS_opto_ given 30 min prior to learning did not affect object exploration during training in SOM-Cre-Raptor-KO mice.

TBS_opto_ given prior to training in SOM-Cre-Raptor-KO mice failed to facilitate object location memory tested 24 h later. During memory tests, both control mice and mice that received TBS_opto_ did not spend more time exploring the mobile object (Fig. 3D middle). Preference ratio of mobile/immobile object was not different in mice that received TBS_opto_ relative to control mice (Fig. 3D right). Thus, TBS_opto_ given 30 min prior to training did not result in facilitation object location memory in SOM-Cre-Raptor-KO mice, suggesting that facilitation of object location learning by TBS_opto_ in SOM-Cre-EYFP mice was due to induction of mTORC1-mediated synaptic plasticity at PC-SOM synapses.

Failure to observe facilitating effects of TBS_opto_ was not due to changes in anxiety levels or locomotion since in open field tests (Fig. 3E–G) mice from both groups showed equivalent anxiety levels (Fig. 3F) or locomotion (Fig. 3G).

## Discussion

The main observations of the study were that (i) silencing SOM-INs during training results in impairment of object location memory, indicating that activity of SOM-INs supports object location learning; (ii) TBS_opto_ given 30 min prior to training results in facilitation of object location memory, suggesting that induction of synaptic plasticity at PC-SOM synapses facilitates object location learning; and (iii) TBS_opto_ given 30 min prior to training did not result in facilitating object location memory in SOM-Cre-Raptor-KO mice, suggesting that facilitation of object location learning by TBS_opto_ in SOM-Cre-EYFP mice was due to induction of mTORC1-mediated synaptic plasticity at PC-SOM synapses. Thus, hippocampal somatostatin interneuron activity is required for object location learning, a hippocampus-dependent form of novelty motivated spatial learning that is facilitated by plasticity at PC-SOM synapses.

### SOM cells activity is necessary for encoding object location memory

Our results indicate that SOM-IN activity is necessary during object location learning. SOM-IN activity is also required for linking context to fear. In contextual fear conditioning, inhibition of SOM-INs during the application of the conditioning stimulus (shocks) reduces contextual fear memory, but inhibition during the context exploration does not [2, 3]. In a passive avoidance task, in which mice avoid an attractive context that is associated with an aversive stimulus, inhibition of the OLMa2 sub-population of SOM-INs during training also reduces fear memory [6]. Our results show that SOM-IN activity is not only necessary for associating fear to context, but also for encoding object relations in space in a novelty-driven task that does not involve aversive stimuli. Although we did not provide electrophysiological confirmation of optogenetic inhibition of SOM-INs by Arch activation in the present work, we showed in a previous report with whole-cell recording in slices that optogenetic activation of Arch hyperpolarizes SOM-INs [18]. A paradoxical release of neurotransmitters from Arch-expressing terminals has been reported with prolonged stimulation with yellow light [33], which could potentially have interfered with our behavioral experiments. Additional experiments using the light-gated chloride pump halorhodopsin (eNpHR3.0) which lacks such paradoxical effects would be useful to confirm our results. However, we have previously shown that sustained Arch activation does not affect basal transmission at Schaffer collateral synapses in hippocampal slices [18]. In addition, we found previously that in open-field control experiments, 5 min continuous yellow light activation or Arch inhibition of SOM-INs did not affect mice exploration, anxiety and locomotion [3], suggesting that the Arch-induced impairment of object location learning was due to SOM-IN silencing. Thus, our findings of an essential role of SOM-IN activity in object location learning, specifically in a mismatch novelty paradigm, extend the necessary role of SOM-IN activity in encoding a wide range of aspects of the context and its relation to salient events in mice.

Interestingly, SOM-IN activity after contextual fear conditioning supports memory consolidation [20]. If the same timing to plasticity is involved in non-aversive behavior—such as the object location task—it would be useful to determine if, using the protocols developed in the present study, activation or inhibition of SOM-IN function, either before or after the object location learning phase, affects consolidation of this memory circuit. Also, the role of SOM-INs vary along the dorso-ventral axis of the hippocampus. Inhibiting OLMa2 cells of intermediate CA1 during passive avoidance training had no effect on fear memory. Furthermore, an opposite role was found in the object recognition task, another novelty motivated memory task where the animal learns the features of objects to differentiate familiar and novel objects. Inhibition of dorsal OLMa2 cells during training has no effect on novel object recognition, while inhibition of intermediate OLMa2 cells facilitates novel object recognition [6]. In this context, it will be interesting to examine the role of SOM-INs in object location memory according to their position along the dorso-ventral axis.

### LTP at PC-SOM synapses facilitates object location memory

Our results indicate that object location memory is facilitated by optogenetic induction of long-term potentiation of SOM interneuron excitatory afferents. It is noteworthy that control mice in our three experimental groups (Figs. 1D, 2D, 3D) did not show similar levels of object location learning. The reason for this discrepancy is unclear but may be due to differences in experimental protocols. For example, the timeline of surgical procedures differed in the Arch and hChR2 groups. Viral injection and cannulation were performed in two separate sessions separated by a one-week recovery period for Arch experiments, while viral injection and cannulation were carried out in one session for hChR2 experiments. However, the three groups of control mice showed normal anxiety and locomotion in the open-field test (Fig. 1E–G, 2E–G, 3E–G). Alternatively, the TBS_opto_ control experiments included an additional session with connection to the optic probe without optogenetic stimulation in home cage, and 30 min later the training session (Fig. 1B versus 2B), which may have negatively affected the object location learning during the training session. However, it is important to note that both control groups in SOM-Cre-EYFP and SOM-Cre-Raptor-KO mice showed similar behavior in the object location learning task, i.e. sub-threshold for learning, validating the comparison of the effects of TBS_opto_ between these mice. The results that facilitation of object location learning was absent in SOM-Cre-Raptor-KO mice indicates that facilitation of object location learning by TBS_opto_ requires mTORC1 function in SOM-INs, suggesting that LTP at PC-SOM synapse positively regulates object location learning.

Previous work indicates that TBS_opto_ given before contextual fear conditioning has the opposite effect and negatively regulates contextual fear memory [3]. In addition, optogenetic induction of PC-SOM synapse LTP prior to contextual fear conditioning, leads to a reduction of subsequent contextual fear conditioning-induced LTP at PC-SOM synapses [3]. In slices, TBS_opto_ given 15 min prior to chemically inducing PC-SOM synapses LTP blocks the protein synthesis normally produced by the chemical LTP induction [34], strengthening the idea that TBS_opto_ interacts with later LTP induction. Interestingly, passive avoidance learning induces long-term potentiation in a subset of pyramidal neurons and this learning-induced potentiation occludes subsequent high frequency stimulation-induced LTP [35]. Thus, an analogous situation may occur in SOM-INs, with induction of LTP by TBS_opto_ at PC-SOM synapses preventing and even reducing LTP induced subsequently during contextual fear conditioning, leading to a deficit in contextual fear memory [3].

Hence the question of why TBS_opto_-induced LTP has different effects on contextual fear and object location memory, when SOM-IN activity is required for both contextual fear and object location learning? An explanation could be that depotentiation of PC-SOM synapses may be required for object location learning. This may be analogous to plasticity at pyramidal cell synapses, where object location learning generally induces long-term depression at Schaffer collateral synapses onto pyramidal cells [36]. LTD of SC-PC synapses may passively propagate to PC-SOM synapses [37] and negatively affect metaplasticity of Schaffer collateral synapses of the CA1 network during learning [3]. Thus, it would be interesting to examine how long-term depression at PC-SOM synapses regulates plasticity of CA3 and entorhinal inputs to pyramidal cells, and how it is influenced by prior induction of TBS_opto_.

However in pyramidal cells, the saturation of hippocampal LTP impairs spatial learning in the Morris water maze task [38]. Also, in mice with cell-specific impairment of mTORC1 activity in SOM-INs, basal synaptic transmission is normal, but LTP is impaired at PC-SOM synapses, and spatial memory in the Barnes maze is deficient. Conversely, in mice with cell-specific facilitation of mTORC1 activity in SOM-INs, LTP at PC-SOM synapses and spatial memory in the Barnes maze are increased [19]. Thus, LTP at PC-SOM synapses appears to be necessary for spatial learning. Consequently, a more plausible explanation for the opposite effect of TBS_opto_ on contextual fear and object location memory, may be that in the present control conditions object location learning was subthreshold for the consolidation of memory, and therefore, long-term plasticity at PC-SOM synapses was likely not induced by learning in these control training conditions. In such “weak” training conditions, prior induction of PC-SOM synapse LTP by TBS_opto_ may not lead later to occlusion of learning-induced long-term plasticity at PC-SOM synapses but to facilitation, resulting in memory formation. Hence, induction of PC-SOM synaptic plasticity by contextual fear and spatial learning may differ, resulting in different sensitivity to occlusion. It will be important in future experiments to determine the plasticity mechanisms induced by spatial learning at PC-SOM synapses. Given that we observed TBS_opto_-induced facilitation of object location learning in "weak” training conditions, it would be important to test if TBS_opto_ also induces a facilitation of object location learning in “stronger” training conditions (i.e. that induce learning), to clarify the role of PC-SOM plasticity in spatial learning. Since learning of novel object location involves an interplay of LTD, LTP, and metaplasticity at CA1 PC synapses [23, 25], it would also be pertinent to examine how LTP at PC-SOM synapses modulates CA1 network metaplasticity induced by novel object location. Whether synaptic plasticity of PC-SOM synapses also regulates other novelty mismatch learning paradigms [23] is another interesting question to address.

Our results of facilitation of object location memory by TBS_opto_ suggests that LTP at PC-SOM synapse regulates hippocampal spatial memory. Because TBS_opto_ was given in vivo, it may have stimulated other synaptic targets of CA1 pyramidal cells, such as subicular neurons, potentially resulting in plasticity at these other output synapses and influencing hippocampal memory [39–41]. To address this possibility, the effect of TBS_opto_ was examined in mice with conditional knock-out of *Rptor* in SOM-INs in which mTORC1-mediated LTP at PC-SOM synapses is blocked [3]. The finding that facilitation of object location memory is absent in these mice, suggests that facilitation of object location memory was due to TBS_opto_-induced plasticity in SOM-INs, and not to actions via other synaptic targets of CA1 pyramidal cells. The finding is also consistent with previous work indicating that, in parvalbumin expressing interneurons, which are another synaptic target of CA1 pyramidal cells, TBS_opto_ does not induce LTP at PC-parvalbumin cell synapses [3]. Interestingly, a subset of the subicular neurons targeted by CA1 pyramidal cells, project back to CA1 pyramidal neurons. Moreover, this feedback from the subiculum is important for formation of object location memory and regulation of pyramidal cell place fields, but not for object recognition [42]. Furthermore, these subicular neurons that feedback to CA1 are inhibited by CA1 SOM-INs [42], likely SOM projection cells [1]. It will be interesting to determine if the synaptic inputs of these SOM projection cells are potentiated by TBS_opto_ and if so, how this regulates the subicular-CA1 network and hippocampal memory. Finally, another useful point in future experiments may be to relate the degree of pyramidal cell recruitment, perhaps visualized with immediate early gene expression, with the TBS_opto_-induced changes in behavioral performance. Such experiments would provide important information about network changes associated with PC-SOM synaptic plasticity and learning.

### Limitations

Our findings are consistent with previous demonstrations that the optogenetic LTP induction protocol (TBS_opto_) in vitro elicits specific long-term potentiation at PC-SOM synapses during whole-cell recordings from SOM-INs in slices, and in vivo regulates contextual fear learning-induced LTP at PC-SOM synapses [3]. However, a caveat of our work is that we did not provide direct proof that the TBS_opto_ induces LTP in vivo. Hence, it would be important to develop an in vivo assay for LTP at PC-SOM synapses to confirm directly the optogenetic induction of LTP in vivo [3]. In addition, it is unlikely that the effects of TBS_opto_ in the present study were due to light-generated heat effect since previous whole-cell experiments in slices have shown that the same TBS_opto_ given to control slices from mice with pyramidal cell expression of mCherry (and no hChR2) did not affect transmission at PC-SOM synapses, and neither did TBS_opto_ given to hChR2-expressing pyramidal cells in the presence of a mGluR1a antagonist, or in mice with a conditional knockout of *Rptor*, an essential component of mTORC1, in SOM-INs [3]. Moreover, in previous behavioral experiments, TBS_opto_ given to mice expressing mCherry (and no hChR2) in pyramidal cells showed normal contextual fear learning compared to unstimulated control mice [3]. Finally, in the present study, TBS_opto_ did not facilitate object location learning in mice with a conditional knockout of *Rptor* in SOM-INs (Fig. 3), suggesting that TBS_opto_ facilitation of object location memory in control mice was unlikely to be due to light-generated heat effects.

A second caveat of our study is the use of mice with global conditional knock-out of *Rptor* in SOM cells that will impair mTORC1 function in SOM cells in other non-hippocampal brain regions and may result in non-specific behavioral changes. However previous work has shown that object location memory is critically dependent on dorsal CA1 hippocampus, compared to other brain regions [26–28]. In addition, these mice display normal exploratory behavior, anxiety level and locomotion in the open field test (Fig. 3), intact sensorimotor gating measured during fear conditioning, and intact non-hippocampal memory such as auditory-cued fear memory [3, 19]. Hence, the effect of the global conditional deletion of *Rptor* in SOM cells is most likely due to interfering with hippocampal SOM-IN mTORC1 function rather than non-hippocampal effects. However, regional- and cell-specific conditional deletion of *Rptor* would be useful for confirmation.

## Supplementary Information

**Additional file 1: Table S1.** Statistical tests details.

**Additional file 2:** DeepLab Cut analysis code: “time in OZ script”.

## Data Availability

The datasets used and/or analyzed during the current study are available from the corresponding author on reasonable request.

## Abbreviations

EYFP: Enhanced yellow fluorescent protein
LTD: Long-term depression
LTP: Long-term potentiation
mGluR1a: Metabotropic glutamate receptor 1a
mTORC1: Mechanistic target of rapamycin complex 1
OLMa2: Oriens lacunosum-moleculare interneurons that express the nicotinic a2 subunit
PC: Pyramidal cells
PC-SOM synapse: SOM-IN excitatory input synapses from local pyramidal cells
SOM-IN: Somatostatin expressing interneuron
TBS: Theta burst stimulation
TBS_opto_: Optogenetic theta burst stimulation
