# Supplementary material for: Object location learning in mice requires hippocampal somatostatin interneuron activity and is facilitated by mTORC1-mediated long-term potentiation of their excitatory synapses
Source: Mol Brain. 2022 Dec 21;15:101. doi: 10.1186/s13041-022-00988-7 (PMC9769025; doi:10.1186/s13041-022-00988-7)
Supplement: Supplementary file 1 — Additional file 1: Table S1. Statistical tests details. [file 13041_2022_988_MOESM1_ESM.pdf]

Supplemental table 1: statistical tests details

Figure	*Variable*	*Group:size*	*Test*	*Mean± sem*	*Statistic*	*Pvalue*
1 D	OLM total exploration time (s) training	EYFP L:7<br>Arch U:8<br>Arch L:8	One way ANOVA	EYFP L 46.6 ± 11.76<br>Arch U 49.4 ± 8.9<br>Arch L 50.3 ± 13.5	F=0.0269	P=0.973
1 D	OLM exploration time (%)	EYFP L:7<br>Arch U:8<br>Arch L:8	Two way repeated measures ANOVA<br><br>Bonferroni pairwise multiple comparison	Training:<br>EYFP L 50.64 ± 5.47<br>Arch U 46.31 ± 5.12<br>Arch L 53.38 ±5.47<br><br>Test:<br>EYFP L 72.54 ± 5.47<br>Arch U 72.07 ± 5.12<br>Arch L 49.9 ± 5.47	Training/Test<br>F=7.876<br>P=0.011<br>Groups x training/test<br>F=3.024<br>P=0.07	EYFP Train vs test p=0.029<br>Arch U Train vs test p=0.008<br>Arch L Train vs test P=0.712<br><br>Training:<br>EYFP vs Arch U p=1<br>EYFP vs Arch L p=1<br>Arch U vs Arch L p=0.706<br><br>Test:<br>EYFP vs Arch U p=1<br>EYFP vs Arch L p=0.013<br>Arch U vs Arch L p=0.012
1 D	OLM test preference ratio	EYFP L:7<br>Arch U:8<br>Arch L:8	Kruskal-Wallis One Way ANOVA on ranks<br><br>Multiple comparison Dunn's method	EYFP L 3.63 ± 1.2<br>Arch U 3.55 ± 0.91<br>Arch L 1.27 ± 0.655	P=0.011	EYFP vs Arch L Q=2.634 P<0.05<br>EYFP vs Arch U Q=0.133 P>0.05<br>Arch U vs Arch L Q=2.588 P<0.05
1 F	OF % time in periphery	EYFP L:7<br>Arch U:8<br>Arch L:8	One way ANOVA	EYFP L 100.35 ± 8.6<br>Arch U 86.07 ± 3.9<br>Arch L 87.13 ± 3.32	F= 1.718<br><br>Power analysis sample size to reach significance overall n= 66	P=0.211

1 F	OF % time in center	EYFP L:7 Arch U:8 Arch L:8	One way ANOVA	EYFP L 3.52 ± 0.77 Arch U 1.72 ± 0.55 Arch L 2.71 ± 0.53	F=1.971 Power analysis sample size to reach significance overall n= 57	P=0.172
1 F	OF ratio time in center/periphery	EYFP L:7 Arch U:8 Arch L:8	One way ANOVA	EYFP L 0.035± 0.006 Arch U 0.02 ± 0.006 Arch L 0.032± 0.006	F=1.658 Power analysis sample size to reach significance overall n= 69	P=0.222
1 G	Total distance	EYFP L:7 Arch U:8 Arch L:8	Kruskal-Wallis One way ANOVA on Ranks	EYFP L 57.65 ± 1.06 Arch U 49.45 ± 1.43 Arch L 56.31±0.5	H=2.025 Power analysis sample size to reach significance overall n= 201	P=0.363
1 G	Zone transitions	EYFP L:7 Arch U:8 Arch L:8	One way ANOVA	EYFP L 620.43±166 Arch U 380.83±100.5 Arch L 578±111.07	F=0.894 Power analysis sample size to reach significance overall n= 93	P=0.429

2 D	OLM total exploration time (s) training	Ctrl :10<br>TBS_{opto} :14	t-test	Ctrl 131.94 ± 32.4<br>TBS_{opto} 112.71 ± 17.22	.t=-0.566	P=0.577
2 D	OLM exploration time (%)	Ctrl :10<br>TBS_{opto} :14	Two way repeated measures ANOVA<br><br>Bonferroni pairwise multiple comparison	Training:<br>Ctrl 50.2 ± 3.114<br>TBS_{opto} 48.36 ± 2.631<br><br>Test:<br>Ctrl 48.8 ± 3.114<br>TBSopto 61.48 ± 2.631	Ctrl/ TBS_{opto} x training/test<br>F = 8.65<br>P = 0.008	Ctr Train vs test p=1<br>TBS_{opto} Train vs test p=0.003<br><br>Training:<br>Ctrl vs TBS_{opto} p=1<br>Test:<br>Ctrl vs TBS_{opto} p=0.02
2 D	OLM test preference ratio	Ctrl :10<br>TBS_{opto} :14	Mann-Witney Rank Sum Test	Ctrl 1.07 ± 0.194<br>TBS_{opto} 2.221 ± 0.63	T=84	P=0.018
2 F	OF % time in periphery	Ctrl :10<br>TBS_{opto} :14	Mann-Witney Rank Sum Test	Ctrl 65.76 ± 6.97<br>TBS_{opto} 76.12 ± 4.64	T=106.5	P=0.292
2 F	OF % time in center	Ctrl :10<br>TBS_{opto} :14	Mann-Witney Rank Sum Test	Ctrl 4.918 ± 1.22<br>TBS_{opto} 5.95 ± 1.71	T=123	P=0.930
2 F	OF ratio time in center/periphery	Ctrl :10<br>TBS_{opto} :14	Mann-Witney Rank Sum Test	Ctrl 0.089 ± 0.025<br>TBS_{opto} 0.101 ± 0.042	T=130.5	P=0.77
2 G	Total distance	Ctrl :10<br>TBS_{opto} :14	Mann-Witney Rank Sum Test	Ctrl 40.81 ± 0.4<br>TBS_{opto} 42.19 ± 0.15	T=125.5	P=0.482
2 G	Zone transitions	Ctrl :10<br>TBS_{opto} :14	t-test	Ctrl 327.5 ± 37.85<br>TBS_{opto} 385.71 ± 24.8	.t=-1.344	P=0.193
3 D	OLM total exploration time (s) training	Ctrl :9<br>TBS_{opto} :10	t-test	Ctrl 144.6 ± 27.7<br>TBS_{opto} 103.9 ± 15.6	.t=1.311	P=0.207

3 D	OLM exploration time (%)	Ctrl :9<br>TBS_{opto} :10	Two way repeated measures ANOVA<br><br>Bonferroni pairwise multiple comparison	Training:<br>Ctrl 50.57 ± 3.077<br>TBS_{opto} 50.49 ± 2.919<br>Test:<br>Ctrl 46.79 ± 3.077<br>TBS_{opto} 56.94 ± 2.919	Ctrl/ TBS_{opto}x training/test<br>F = 5.448<br>P = 0.032	Ctrl: train/test p=1<br>$TBS_{opto\,:}$ train/test p=0.284<br>Training: ctrl/ TBS_{opto} p=1<br>Test: ctrl/ TBS_{opto} p=0.143
3 D	OLM test Preference ratio	Raptor U:9<br>Raptor L :10	t-test	Ctrl 0.957 ± 0.137<br>TBS_{opto} 1.482 ± 0.218	.t= -1.986	P=0.063
3 F	OF % time in periphery	Raptor U:9<br>Raptor L :10	Mann-Witney Rank Sum Test	Ctrl 76.22 ± 7.315<br>TBS_{opto} 77.7 ± 5.95	T=87	P=0.838
3 F	OF % time in center	Raptor U:9<br>Raptor L :10	t-test	Ctrl 3.31 ± 0.63<br>TBS_{opto} 2.87 ± 0.48	.t=0.555	P=0.586
3 F	OF ratio time in center/periphery	Raptor U:9<br>Raptor L :10	Mann-Witney Rank Sum Test	Ctrl 0.51 ± 0.013<br>TBS_{opto} 0.38 ± 0.005	T= 96	P=0.653
3 G	Total distance	Raptor U:9<br>Raptor L :10	t-test	Ctrl 38.44 ± 0.4<br>TBS_{opto} 43.83 ± 0.33	.t= -1.046	P=0.31
3 G	Zone transitions	Raptor U 9<br>Raptor L 10	t-test	Ctrl 317.78 ± 27.28<br>TBS_{opto} 370.8 ± 33.66	.t=1.206	P=0.244

Abbreviations:

OLM: Object location memory U: Unlit

OF: Open field L: Light
