# Supplementary material for: Object location learning in mice requires hippocampal somatostatin interneuron activity and is facilitated by mTORC1-mediated long-term potentiation of their excitatory synapses
Source: Mol Brain. 2022 Dec 21;15:101. doi: 10.1186/s13041-022-00988-7 (PMC9769025; doi:10.1186/s13041-022-00988-7)
Supplement: Supplementary file 2 — Additional file 2: DeepLab Cut analysis code: “time in OZ script”. [file 13041_2022_988_MOESM2_ESM.pdf]

# Put this script in the folder with deeplabcut's csv results files, it will open all csv files in this folder. Then you can choose to directly print the results in your prompt, the script will then print the file name taken from the deeplabcut results csv which is the same as the video it summarizes, and then the results. I called my videos by the test session (acq for training and tst for test) and the animals number so in the end I had: acq or tst, animal number, time in IOZ xx time in MOZ xx; with I/M OZ being Immobile/Mobile object zone. The other option is to print the results in a csv, to build this csv, I took the test session (acq or tst) mice number from the deeplabcut result csv name. So for this script to work you will need to call your videos the same way or to change the script (I will show you where).

```
import pandas as pd

import os

import re

import csv


if not os.path.exists('results'):

    os.mkdir('results')


def isInside(IOZ_x, IOZ_y, rad, x, y):

    # Compare radius of circle with distance of its center from given point to tell if the animal's head is
    within the 3 cm radius circular zone around the objects.

    if ((x - IOZ_x) * (x - IOZ_x) +

        (y - IOZ_y) * (y - IOZ_y) <= rad * rad):

        return 1

    else:

        return 0

results = {}

list_csv = os.listdir()

for filename in list_csv:

    if not filename.endswith('.csv'):

        continue

    df = pd.read_csv(filename, header=[1,2])

    print(filename)


    df.astype("float")
```

```
# This defines the objects positions
IOZ_x = df['imobilobj']['x'].mean()
IOZ_y = df['imobilobj']['y'].mean()
MOZ_x = df['movedobj']['x'].mean()
MOZ_y = df['movedobj']['y'].mean()
rad = 75 # nb of pixels = to 3 cm in my arena.


df["insIOZ"] = df.apply(lambda row: isInside(IOZ_x, IOZ_y, rad, row['head']['x'], row['head']['y']), axis=1)
df["insMOZ"] = df.apply(lambda row: isInside(MOZ_x, MOZ_y, rad, row['head']['x'], row['head']['y']), axis=1)
# This divide the number of frames where the animal's head is in the zone by the video speed to have the time spent in the zone
insIOZtime = df['insIOZ'].sum()/30 #30 is the nb of frame/s in my videos, change it according to your videos speed.
insMOZtime = df['insMOZ'].sum()/30 # and here
# To print directly in powershell
# print(f'time in IOZ {insIOZtime}')
# print(f'time in MOZ {insMOZtime}')
#  print()


# print these results in results folder output csv: mouse; acq immobile object; acq mobile objects; test immobile object; test mobile object.
test_name = re.search(r'tst[0-9]', filename) # If you did not put tst in the test video name, change it here
if test_name:
    mouse = test_name.group(0).replace('tst', 'm') # and here, and maybe m ( because I called my mice m1,m2…)


    if mouse not in results:
        results[mouse] = {}
    results[mouse]['test'] = [insIOZtime, insMOZtime]
```

```
    acq_name = re.search(r'acq[0-9]', filename) # If you did not put acq in the training video name, change
it here
    if acq_name:
        mouse = acq_name.group(0).replace('acq', 'm') #and here
         if mouse not in results:
           results[mouse] = {}
          results[mouse]['acquisition'] = [insIOZtime, insMOZtime]


    if not test_name and not acq_name:
        print('Huh oh')


# print(results)
header = ['mouse','acq immobile', 'acq mobile', 'test immobile', 'test mobile']


with open('results/output.csv', 'w', newline='') as f:
# create the csv writer
    writer = csv.writer(f)
# write a row to the csv file
    writer.writerow(header)
    for mouse in results:
          writer.writerow([mouse, results[mouse]['acquisition'][0], results[mouse]['acquisition'][1],
results[mouse]['test'][0],  results[mouse]['test'][1]])
```
